# Normal colon epithelium: a dataset for the analysis of gene expression and alternative splicing events in colon disease

**DOI:** 10.1186/1471-2164-11-5

**Published:** 2010-01-04

**Authors:** Wilfrido Mojica, Lesleyann Hawthorn

**Affiliations:** 1 Department of Pathology, University at Buffalo, SUNY, Buffalo, NY, USA; 2 Molecular Oncology Program, Medical College of Georgia Cancer Center, Augusta, GA, USA

## Abstract

**Background:**

Studies using microarray analysis of colorectal cancer have been generally beleaguered by the lack of a normal cell population of the same lineage as the tumor cell. One of the main objectives of this study was to generate a reference gene expression data set for normal colonic epithelium which can be used in comparisons with diseased tissues, as well as to provide a dataset that could be used as a baseline for studies in alternative splicing.

**Results:**

We present a dependable expression reference data set for non-neoplastic colonic epithelial cells. An enriched population of fresh colon epithelial cells were obtained from non-neoplastic, colectomy specimens and analyzed using Affymetrix GeneChip EXON 1.0 ST arrays. For demonstration purposes, we have compared the data derived from these cells to a publically available set of tumor and matched normal colon data. This analysis allowed an assessment of global gene expression alterations and demonstrated that adjacent normal tissues, with a high degree of cellular heterogeneity, are not always representative of normal cells for comparison to tumors which arise from the colon epithelium. We also examined alternative splicing events in tumors compared to normal colon epithelial cells.

**Conclusions:**

The findings from this study represent the first comprehensive expression profile for non-neoplastic colonic epithelial cells reported. Our analysis of splice variants illustrate that this is a very labor intensive procedure, requiring vigilant examination of the data. It is projected that the contribution of this set of data derived from pure colonic epithelial cells will enhance studies in colon-related disease and offer a vital baseline for studies aimed at elucidating the mechanisms of alternative splicing.

## Background

Colorectal cancer (CRC) is considered to develop as a result of a multi-step progression and accumulation of genetic alterations [1]. Gene expression profiling using microarrays has been used extensively in attempts to identify the genetic events associated with this progression. For colon cancer, expression profiling can readily discriminate between tumor and normal tissue as well as distinguish between tumors of different histopathological stage and prognosis to some extent [2,3]. It is anticipated that the identification of these gene expression differences may lead to a better understanding of neoplasia, help identify diagnostic and prognostic biomarkers, and lead to the discovery of new therapeutic targets. As with any microarray study, however, tissue heterogeneity is a confounding factor. Previous studies on CRC have often used "adjacent normal colon" as a control cell type. However, this tissue usually represents a mixture of cell types which do not accurately represent the epithelial cell lining of the luminal surface of the colon that gives rise to CRC. Clearly, a comprehensive and representative gene expression profile exclusively from normal colon epithelial cells would be a great benefit for comparison with tumor cells, especially since these cells are not readily cultured.

Here we report a reliable expression reference data set for non-neoplastic colonic epithelial cells. These cells were derived from fresh, unfixed, clinically excised non-tumor related colectomy specimens and enriched using magnetic beads coated with a colon cell specific antibody. RNA from these preparations was used to probe Affymetrix GeneChip^® ^EXON 1.0 ST arrays. These arrays not only report gene expression levels but can also identify differential splicing events.

The findings from this study represent the first comprehensive expression profile for non-neoplastic colonic epithelial cells. We have compared the data derived from these colonic epithelial cells to a publically available set of tumor and normal matched colon data that had been hybridized to the same arrays. The dataset is available from Affymetrix.com http://www.affymetrix.com/support/technical/sample_data/exon_array_data.affx. This analysis allowed an assessment of global gene expression alterations in the tumors and demonstrated that adjacent normal tissues are not always the optimal control.

Alternative splicing (AS) is the process by which the exons of primary transcripts (pre-mRNAs) can be spliced in different arrangements to produce structurally and functionally distinct mRNA and protein variants. Alternative splicing leads to massive diversity by generating multiple different mRNAs from a single template. Current estimates indicate that up to 95% of genes in the human genome exhibit alternative splicing [4]. Furthermore, alternative splicing has increasingly been acknowledged as critical during normal development. Previous studies of alternative gene splicing have, for the most part, focused on the identification and characterization of the control of splice site selection on a gene-by-gene basis. While these studies have provided extensive information about the factors and interactions that control formation of the spliceosome, relatively little is known about the global regulatory properties of alternative splicing [5,6].

In this analysis, we also compared AS events in tumors and the enriched normal colon epithelial cells. This analysis of splice variants on a genome-wide scale, however, is very labor intensive, and requires careful supervised examination of the data. It is anticipated that the contribution of this set of data derived from pure colonic epithelial cells will facilitate future studies in colon-related disease and offer a unique baseline for studies aimed at elucidating the mechanisms of alternative splicing.

## Results

One of the main objectives of this study was to generate a reference gene expression data set for normal colonic epithelium which can be used in comparisons with tumor samples. Non-adenomatous and non-neoplastic colonic epithelial cells were procured from 10 different colectomy specimens with the clinical indication for each case being diverticular disease. Of the 10 patients, 6 were female and 4 male, with the ages ranging from 47 to 80 (table 1). The surgeries were performed by 6 different surgeons with four of the specimens excised by hand assisted laparoscopy and the other 6 performed by the conventional open surgical approach. The procurement protocol was designed to effectively enrich for the selected colonic cell population in the shortest time possible to control for variables that may influence the quality and integrity of clinical biospecimens, as described in the Materials and Methods section. The data discussed in this publication have been deposited in NCBI's Gene Expression Omnibus and are accessible through GEO Series accession number GSE19163 http://www.ncbi.nlm.nih.gov/geo/query/acc.cgi?acc=GSE19163

**Table 1:** Clinical Correlates of Patient Cohort

Case #	Age	Sex	Location	Surgical Approach	Diagnosis
1**	55	F	Sigmoid	Resection	Diverticulitis

2	58	M	Sigmoid	Resection	Diverticulosis

3	67	M	Sigmoid	Laparoscopic	Diverticulosis

4**	58	M	Sigmoid	Laparoscopic	Diverticulosis

5	62	M	Sigmoid	Laparoscopic	Diverticulitis

6	80	F	Sigmoid	Resection	Diverticulosis

7	47	F	Sigmoid	Resection	Diverticulosis

8	65	F	Sigmoid	Resection	Diverticulosis

9	62	F	Sigmoid	Resection	Diverticulosis

10	58	F	Sigmoid	Laparoscopic	Diverticultis

Demographics of patients from where the enriched samples of non-neoplastic colonic epithelial cells were derived. ** Indicate those samples on which replicates were performed.

### Gene Expression

**Figure 1:**
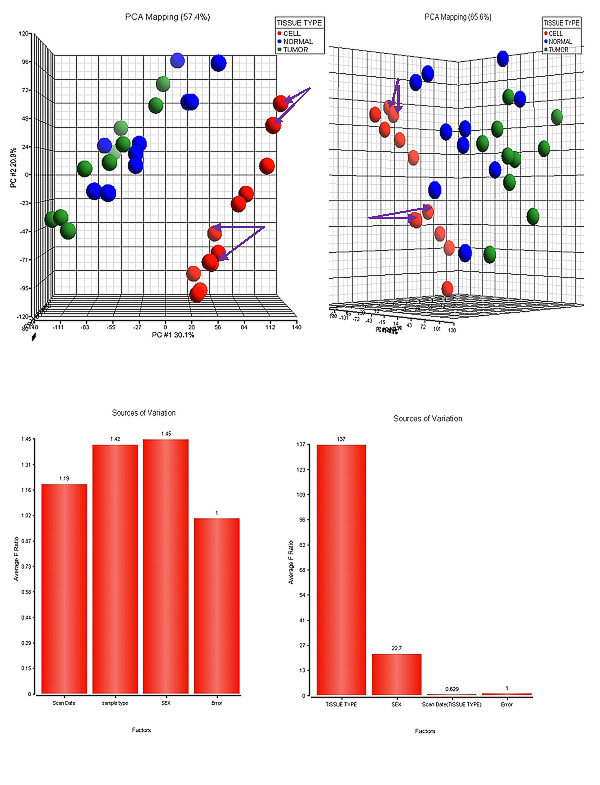
**Principle Components Analysis and Analysis of Variance of Gene Expression Data**: The individual genes are summarized from exon intensities mapping to each locus. The PCA plot shows data from CELLS (red), NORMAL tissues (blue) and tumors (green). It can be seen that the tumor and normal tissues cluster together while the CELLs form a discreet cluster distant from the other samples. (B) Following a 3-way ANOVA the Sources of Variation were plotted. It can be seen that "SCAN DATE" is a major contributer to the variation. This can be attributed to the variation in processing performed by the 2 different laboratories. Also seen as a major source of variation is SEX. This is due to the fact that 7 of the samples in the CELL group were classified as female whereas the TUMOR/NORMAL set of data had 5 of each sex. (C) The SEX and SCAN DATE sources of variation were removed from the ANOVA analysis and the PCA performed. It can be seen that the three sample types cluster more closely but the CELLs still retain a degree of separation. (D) shows the ANOVA -Source of Variation histogram following the removal of the batch effects due to SCAN DATE and SEX. It is now notable that the major source of variation is due primarily to the different TISSUE TYPEs.

Twelve samples of isolated colonic epithelial cells from 10 different patients (two of these samples were replicates; see table 1 and figure 1a) were analyzed using the Exon 1.0 ST arrays. These data were then compared with a publically available data set http://www.affymetrix.com/support/technical/sample_data/exon_array_data.affx, [7] which provided raw Exon1.0 ST data for ten colon tumors and matched 'normal 'tissues from the same patients. Total RNA from these samples were purchased from Biochain Institute Inc. All of the raw data was imported into PARTEK and the arrays normalized using quantile normalization followed by probeset summarization using RMA. PCA analysis for these data sets is shown in figure 1a, where it is evident that the NORMAL and TUMOR tissues from the public data set clustered together, while the CELL samples obtained from our enrichment procedure separated distinctly from the two other tissue sources. A 3-way ANOVA identified that a major source of variance was due to the sample processing differences between the 2 laboratories, referred to as "*SCAN DATE*" (Figure 1b). Patient "*SEX*" was also noted as a major source of variance which was primarily due to the fact that the tumor/normal colon data set consisted of 5 males and 5 females, while our analysis used samples from 7 females and 3 males which slightly skewed the analysis for this category. To correct for these batch effects, therefore, we removed two of these sources of variance and reanalyzed the data where it is clear that the 3 types of tissues now cluster more closely (Figure 1c) although the CELLs still form a discrete cluster while the TUMOR and NORMAL tissues cluster together in no defined pattern. Figure 1d displays the ANOVA histogram showing that the variation attributable to SCAN DATE and SEX have been removed.

**Figure 2:**
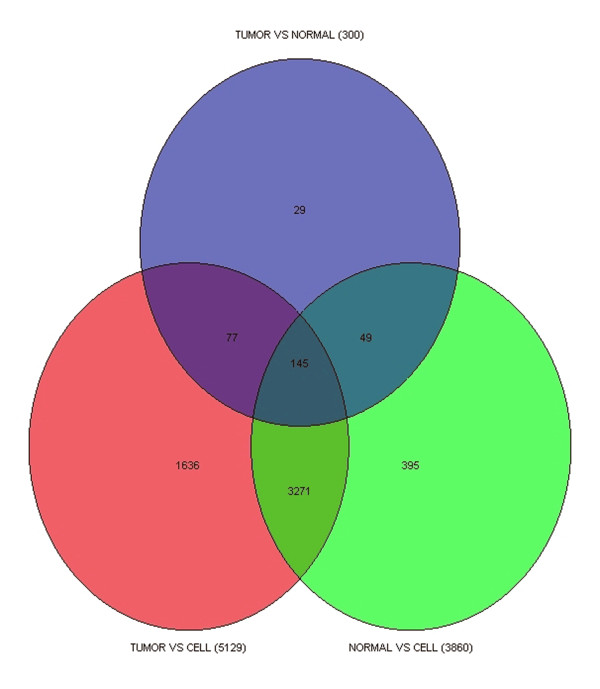
**Venn Diagram of gene expression alterations for three comparisons**. The tumor vs cell comparison showed the largest number of transcript expression changes, while the tumor compared to the adjacent normal tissues showed the lowest number. The 77 genes which show alterations in both the tumor vs cell and tumor vs normal are of interest. All comparisons were performed following RMA normalization of all raw image files. Following a 3-way ANOVA the sources of variation were removed and the comparisons conducted. Cut-off values for gene expression differences were p = 0.05 and combined with a > 2-fold change.

Following this corrective processing, the various cut-off values for expression differences were set at p = 0.05 for the experimental sets compared to normal and combined with fold change values of 2. Genes showing differential expression using these analysis parameters are shown in the Venn diagram in Figure 2. This analysis reveals that there are more changes in the comparison between the isolated epithelial cells and the tumor than there were in the comparison between the matched tumor and normal tissue. Genes showing the highest increased expression levels in the tumor vs CELL comparison were OLFM4, FN1, ACTG2, IGJ, ACTA2, COL1A2, SPARC, RCN1, COL3A1 and COL1A1. The genes with the most significant decreases included TMIGD1, OTOP2, CA4, ZG16, MS4A12, GUCA2B, BEST4, CD177, HRASLS2, IFIT1 and MUC17.

The data was then analyzed using Ingenuity Pathways Analysis (IPA version 7.5). One of the top canonical pathways associated with the DEG list generated from the comparison of tumor to CELLs was the WNT Signaling Pathway. Fifty eight genes from our analysis of tumor compared to CELLs were deregulated in this pathway consisting of 160 members in total (p = 7.81E-07. The pathway is shown in Additional File 1 and it is noteworthy that many of the members of this pathway are up-regulated in tumor tissues. A network generated from the data analysis is shown in Additional file 2. This network involves Cancer Cell Cycle, Cellular Growth and Proliferation functions with an associated score of 20 (p = 10E-20) and has 35 molecules fitting into the network. It is apparent from the network ideogram that all molecules interact either directly or indirectly through the CDKN1A gene.

The most significant biological function associated with disease and disorder category was Cancer with 1126 of the molecules in the comparison of tumors to epithelial cells fitting into this category with an associated p-value of 3.02E-35. The most significantly altered subcategories within this functional category included; Tumorigenesis (731 molecules from the DEG list, p = 3.02E-35), Neoplasia (714 molecules, p = 8.98E-34), Cancer (691 molecules, p = 3.83E33) Colorectal Cancer (256 molecules, p = 2.56E-26 and Colon Cancer (161 molecules, p = 6.10E-25)

The most significant (p = 1.45E-21) molecular and cellular function was Cell Growth and Proliferation, a category which describes functions associated with cell growth including colony formation proliferation and outgrowth of cells. Six hundred and thirty nine of the genes from the DEG fit into this cellular function category. The most significant (p = 2.47E-11) physiological function was Tissue Development with 180 genes from the DEG list fitting into this functional category. This category includes functions associated with development and differentiation and the formation of tissues through the association of cells including growth, patterning and survival of tissues, accumulation, adhesion and clustering of cells.

The comparison of tumor to normal tissue showed fewer gene expression differences with much lower fold changes. The highest increases in gene expression included, LGR5, CST1, SPP1, CLDN1, MMP11, INHBA, THBS2 SFRP4, ETV4 and SLC6A6. The most significantly down-regulated genes included SLC26A3, GUCA2A, ZG16, CLCA4, MYH11, AQP8, TMIGD1, CA2, CLCA1, ANPEP and ADH1C.

The most significant (p = 7.69E-07) canonical pathway associated with the DEG list generated from the comparison of tumor to normal tissue was the Hepatic Fibrosis/Hepatic Stellate Cell Activation Pathway. Interestingly, this was also one of the top 5 pathways found associated with the tumor vs CELL comparison. Eleven genes of a possible 126 genes in this canonical pathway were altered in the comparison between tumor and normal tissues. This most significant network generated from this comparison was Cancer, Cell Movement, Hematological System Development and function. The score for this network was 16 (p = 10E-16) and is shown is shown in Additional file 3. It is apparent from the network ideogram that all molecules interact either directly or indirectly through the TNF or IL1B genes, although these genes themselves do not show altered expression in the tumors compared to normal tissues.

The most significant biological function associated with disease and disorder category was Cancer and 128 of the molecules in the comparison of tumors to CELLs fit into this category with an associated p-value of 1.89E-30. The most significant (p = 5.10E-11) molecular and cellular function was Cellular Movement, 46 of the genes from the DEG fit into this cellular function category. The most significant (p = 4.74E-10) physiological function was Tissue Morphology with 22 genes from the DEG list fitting into this functional category. This category includes functions associated with development and differentiation and the formation of tissues through the association of cells including growth, patterning and survival of tissues, accumulation, adhesion and clustering of cells.

Thus, from this analysis, it is clear that the altered gene expression profiles depend on the origin of the normal sample. Despite the variation in the analysis between the tumor and different normal samples, 77 genes showed the same changes regardless of the normal sample used (figure 2). This analysis strategy demonstrates an important application of this data because it verifies the gene expression changes that occur in both isolated cells and hetergenous normal tissue, while omitting the transcript alterations that are attributable to tissue heterogeneity alone. The genes showing the highest expression changes in tumor compared to both normal tissues and CELLs were CLDN1, CST1, KIAA1199, MMP14, RFC3, MTHRD2, ZNF587, MMP11, INHBA and PSAT1. Genes showing the largest decreases in expression included; GUCA2A, KPNA7, SLC26A3, PLAC8, ANPEP, SLC26A2, FABP1, DHRS9, SLC4A4 and TSPAN1.

IPA analysis of the data showed that the canonical pathway with the highest number of members from this list of 77 overlapping genes was the Bladder Cancer Signaling Pathway with a significance value of p = 9.26E-06. Only 5 members of the 77 were deregulated in a pathway consisting of 88 molecules. The WNT Signaling Pathway was also in the top 5 canonical pathways with a p value of 1.56E-03. The lower p values in this comparison is expected because the list of genes is very limited.

The top network generated from this DEG list is Cancer, Cell Growth and Proliferation, Cellular Movement and is shown in Additional file 4. This pathway has an associated score of 30 (p = 10E-30). The majority of the genes showing down-regulation interact with IL8, TNF and TGFB1, while genes showing increased expression show direct interactions with HNF1A.

The biological functional analysis showed that the most significant (p = 5.47E-15) disease and disorder category generated by this list of 77 overlapping genes was Cancer. It is noteworthy this was also the most significant generated by tumor vs CELL comparison. The most significant (p = 8.84E-09) molecular function was Cell Death, a functional category associated with cytolysis, necrosis, survival and recovery of cells. Twenty one genes from the list of 77 fit into this category, of which 14 demonstrated up-regulation. The most significant (p = 7.29E-06) physiological function identified was Tissue Development with 9 molecules fitting in this functional category. Four of these were involved in the accumulation of cells and were all up-regulated in the tumors. An additional 3 up-regulated genes were associated with formation of the endothelial tube and an additional 6 (some genes belong to more than one sub-category) were associated with cell adhesion.

### Exon-Level Analysis

**Figure 3:**
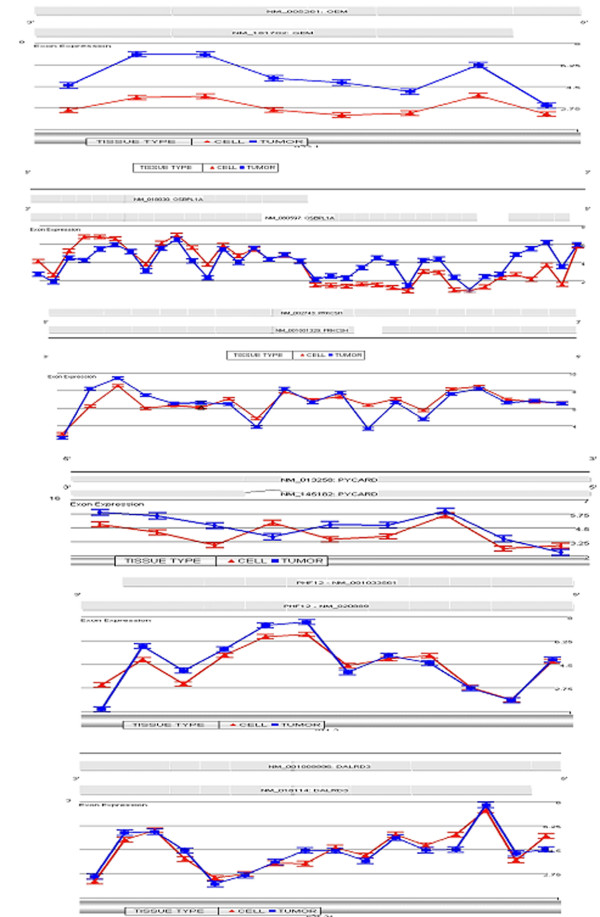
**Alternative Splicing Events in genes known to have AS**. The grey bars along the top of each diagram represent the RefSeq gene annotation. Traces in the upper region of each figure show average intensities of probesets representing exon regions for tumors (blue) and Cells (red). **(a)**Shows an example where the alternative splicing index for the GEM gene has a very low p value indicating alternative splicing events, however the fold change values for the entire transcript are ~5 fold suggesting that this is differential expression **(b) **The tumor tissue is expressing the full length isoform B of the OSBPL1A gene, while the cells are expressing the truncated version, isoform A, which has an alternative 5' start. **(c) **shows a classic example of a cassette exon, where the tumors are expressing isoform 1 of the PRKCSH gene and = the colon epithelial cells are representing isoform 2. **(d) **demonstrates another example of a cassette exon in the PYCARD gene. The third exon is not expressed in the tumor (α-isoform) but is expressed in the cells (β-isoform). **(e) **shows an example 3' alternative splicing in the PHF12 gene and **(f) **displays an example of a 5'splicing event in the DALRD3 gene.

Since the ST arrays not only report gene expression levels but also splicing differences, we reanalyzed the data to identify splice variants between the various samples. A major complication in the analysis of the AS data, however, comes from the differential detection of transcription and mRNA processing. If the hybridization intensities for the individual probesets are examined, and these differences are used as an index of splicing, incorrect conclusions will be drawn because the intensities are not considered within the context of the entire transcript. As shown in figure 3a, the average intensities for the probesets representing the exons of the GEM gene show a high p-value (1.2e-10) for alternative splicing in a comparison between the tumors and CELLs, but the entire transcript has an overall increased expression in the tumors compared to the CELLs (average fold change -5). To overcome this complication, we devised a more strict way of detecting AS, which was designed to decrease the false positive rate, although it may also have eliminated some possible AS events. We also limited our analysis to a comparison between the tumors and isolated epithelial cells (CELLs). The exon-specific probesets required p values for alternative splicing of <0.0001, the p value for whole transcript expression was >0.9 and the fold change cut off for whole transcript expression had to be <2.5 and >-2.5. In this way we eliminated whole transcript expression while detecting exon-specific changes and defined 497 genes showing alternative splicing. Each of these genes was then examined visually using Partek Genomics Suite gene view. The full data set is available in Additonal file 5.

To evaluate the utility of our approach, we selected a subset of genes that were known to be alternatively spliced as recorded in the UCSC Genome Browser http://genome.ucsc.edu/. Upon visual examination many of the genes included in our analysis failed to display convincing AS events in genes known to be alternatively spliced, highlighting the need for more careful examination of the data. However, we found a number of genes that displayed convincing alterative splicing in genes with known AS. Table 2 describes the genes that we have selected. A few examples of these are shown in figures 3b, 3c, 3d, 3e and 3f. The average intensities of the probe sets representing the OSBPL1A gene are shown in figure 3b. The tumor tissue expresses the full length isoform B while the CELLs express the truncated isoform A version. Another example is shown in Figure 3c which displays the average intensities for the probesets representing the exons of the PRKCSH gene. The tumors express isoform 1 of this gene, while the colon epithelial cells typify isoform 2. Figure 3d shows that the overall expression level for PYCARD is higher in the tumor tissue which expresses the α-isoform of this gene, while the CELLs clearly express the β isoform. Figure 3e shows the intensity plot for PHF12 where the tumor expresses the truncated 3' exon and the CELLs express the full length exon. This gene has over 30 different alternative splice variants recorded. An example of alterative 5' exon expression is shown in figure 3f in the DALRD3 gene.

**Table 2:** Alternatively spliced genes in a comparison of tumors and isolated epithelial cells.

# Probesets	Transcript ID	Gene Symbol	RefSeq	Alternative Splicing p-value	Fold Change Tumor vs Cell
13	3959350	APOL3	NM_030644	7.20E-11	-1.0483

14	3079463	ABCF2	NM_007189	2.53E-16	-1.0105

14	3082759	DLGAP2	NM_004745	0.0102642	-1.75766

20	3859761	DMKN	NM_033317	1.72E-25	-1.00705

33	3132016	FGFR1	NM_023111	0	1.86159

17	4026722	IDH3G	NM_174869	3.34E-21	1.37367

12	4027708	MTCP1	NM_001018025	0.000139499	-1.12104

22	2628785	MITF	NM_006722	1.62E-27	1.04061

23	3795184	NFATC1	NM_172389	1.05E-19	1.03091

54	3564071	NIN	NM_020921	7.07E-13	1.03063

35	2517588	OSBPL6	NM_032523	2.44E-10	-1.26328

40	2858134	PDE4D	NM_006203	0	-1.69311

29	3923632	PFKL	NM_001002021	0	1.258

12	3751184	PHF12	NM_001033561	8.89E-18	1.09533

19	3821200	PRKCSH	NM_001001329	0	-1.0575

9	3688311	PYCARD	NM_145182	2.36E-10	1.58277

38	2395245	RERE	NM_012102	0	-1.10969

38	2683763	ROBO1	NM_002941	0	2.52196

25	3930360	RUNX1	NM_001754	0	2.48266

14	4007588	SLC35A2	NM_001042498	3.63E-08	-1.01314

23	2428313	ST7L	NM_138729	1.59E-42	-1.26644

18	3049025	TBRG4	NM_199122	0.00E+00	2.29256

21	2402601	UBXD5	NM_183008	3.82E-14	1.00668

12	2706791	ZMAT3	NM_152240	1.02E-19	1.5543

The genes were selected by limiting the p-values for alternative splicing to <.0001. To eliminate genes that had whole transcript level differences the p value for gene expression was set at p > 0.9 and a fold change of <2.5 or >-2.5. The individual probesets for the genes were then visually examined and those with corresponding reports of alternative splicing events in RefSeq were included in the analysis.

**Figure 4:**
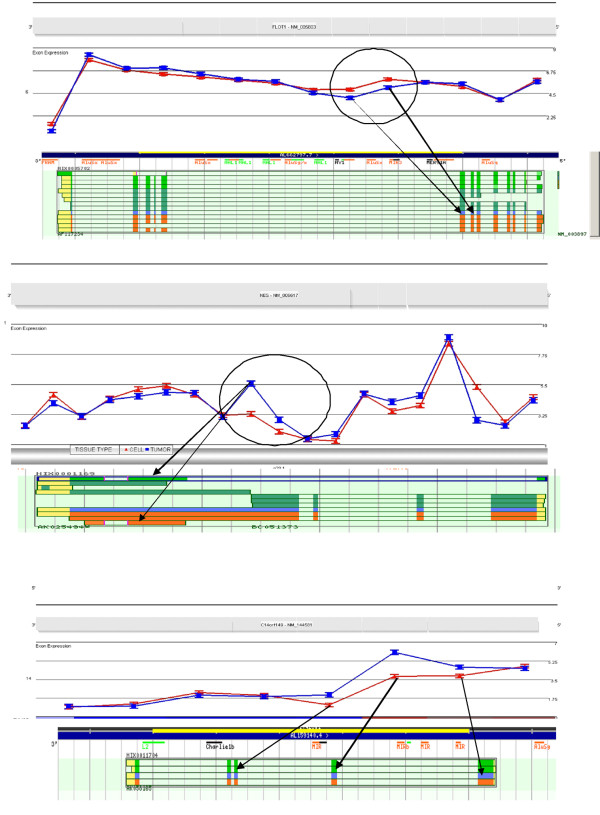
**AS events without RefSeq Reports**. The grey bars along the top of each diagram represent the RefSeq gene annotation. Traces in the upper region of each figure show average intensities of probesets representing exon regions for tumors (blue) and Cells (red). The lower insets show extensive annotatation provided by the H-InvDB. RefSeq annotation is shown in blue and ENSEMBL in orange **(a) **RefSeq annotates a single transcript for the FLOT1 gene while the H-InvDB reports annotation for 7 variants shown in the inset. None of the additional transcripts concur with the profile provided by the array. **(b) **The NES gene shows differential expression of 2 probesets in the 3' exon. RefSeq reports a single variant of this gene, H-InvDB reports 11 variants. The region on the 3' exon that shows higher expression in the tumors corresponds to AS events in the first and last transcripts (shown by arrows) reported by H-InvDB. **(c) **The C14orf149 gene appear to be differentially spliced between the two tissue types at exons 3,4 and part of 5 of. RefSeq details a single transcript for this gene, while H-Inv reports 4 alternatively spliced variants. None of the reported variants explain the differences between the two tissues profiles obtained from the exon array analysis.

Of the 497 genes apparently showing alternative splicing, approximately 60% showed AS events involving exons that had not been reported in RefSeq. These data demonstrate the incompleteness of our current understanding of the extent of AS. For example, (figure 4a) in RefSeq there is only one representative transcript for the FLOT1 gene with no alternative splicing. The H-InvDB (Human Full-length cDNA Annotation Invitational DataBase), released in December 2008 http://www.h-invitational.jp/hinv/ahg-db/index.jsp however, describes 7 variants of FLOT1 (Figure 4a). Interestingly, none of these variants have the same profile of the patterns shown in figure 4a, where the tumor appears to have no expression of 5^th ^and 6^th ^exons from the 3' end of the gene. There are 2 transcripts that are missing those exons but none that have retained the 5' exon. Since this gene shows high expression levels, it is possible that the 5' probes could exceed the saturation threshold and therefore the array is not capable of discriminating a loss at the 5' end.

In the same way, the NES gene (figure 4b) apparently shows differential expression of 2 probe sets in the last 3' exon and, while RefSeq reports a single variant of this gene, H-InvDB reports 11 variants. The RefSeq variant is shown in figure 4b insert in blue. The region on the 3' exon that shows higher expression in the tumors corresponds to the first and last transcripts reported by H-InvDB.

Another interesting example is shown in figure 4c where exons 3, 4 and part of 5 of the C14orf149 gene appear to be differentially spliced between the two tissue types. RefSeq details a single transcript for this gene, while H-Inv reports 4 alternatively spliced variants. There are currently no known variants that would explain the differences in the profiles for the two tissues. Figure 4c shows 10 transcripts reported in H-Inv for the C20orf149 gene, none of which correspond to the probeset which shows differential splicing between the colon epithelial cells and the tumor tissues. The circled transcript closely approximates what is seen on the array but the actual probeset maps within an expressed sequence.

**Figure 5:**
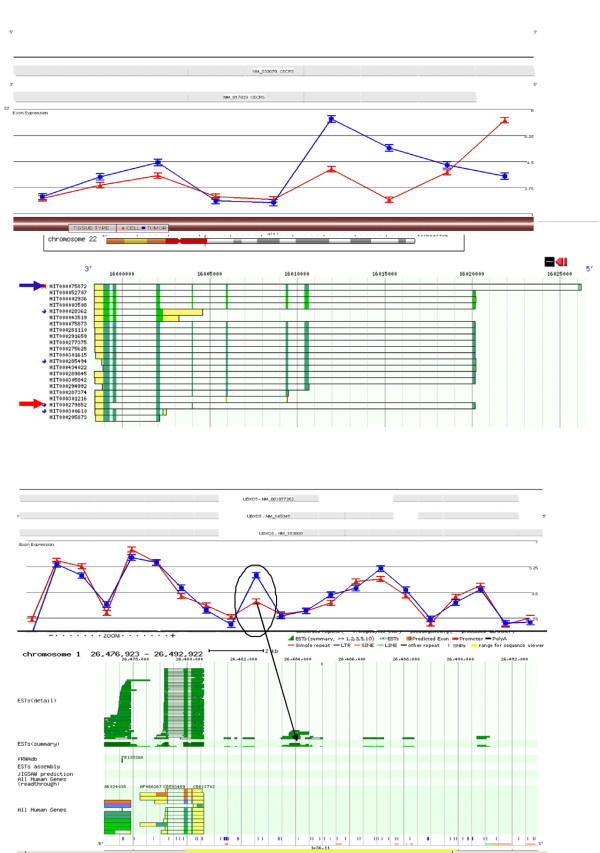
**Alternative Splicing Events in Exons Other Than Those Reported in RefSeq**. The grey bars along the top of each diagram represent the RefSeq gene annotation. Traces in the upper region of each figure show average intensities of probesets representing exon regions for tumors (blue) and Cells (red). The lower insets show extensive annotatation provided by the H-InvDB. **(a) **The trace displays the average intensities for the probe sets representing the exons of the CECR5 gene. Expression differences are evident v for exons 4 and 5 and show higher values for the tumor. The 5' exon also shows differences whereby the CELLs express an alternative 5' isoform which has been reported in RefSeq (grey bars). The H-InvDB reports 27 alternative splicing events. The blue arrow highlights the transcript (HIT00075872) the tumor is expressing with exons 3 and 4 retained but the 5' exon is missing. The CELLs appear to be expressing a transcript with AS events at exons 3, 4, 6 and 7 indicated by the red arrow (HIT000279852. **(b) **The array has a probeset that maps to an intron between exons 9 and 10 in the UBXD5 gene and this region is retained in the tumor samples. The tumor appears to be expressing sequences from the intron between exons 9 and 10. RefSeq annotates 3 representative transcripts, while H-InvDB annotates 15 different transcripts but none of these include retention of this intron. The insert shows a map of the expressed sequenced tag sites ESTs that map to this region on chromosome 1. A collection of these (delineated by the arrow) have been placed in the exact position of the retained intron on UBXD5.

In many cases, the probesets would have alternative splicing events in exons or introns that were not known to be alternatively spliced, although other regions of the genes were reported in RefSeq as AS events. These examples further highlight the complexity of AS. Figure 5a shows the average intensities for the probesets representing the exons of the CECR5 gene. The expression intensities for the last 3 exons are very similar but values for exons 4 and 5 show higher values for the tumor. Exon 2 shows similar intensities between the two tissues and they diverge at the 5' exon where the CELLs show an alternative 5' isoform which has been reported in RefSeq. The H-InvDB reports 27 alternative splicing events (Only a few are shown). The arrow shows the most likely transcript (HIT00075872) whereby the tumors have retained exons 3 and 4 but the 5' exon is missing. The CELLs appear to be expressing the HIT000279852 transcript that is missing exons 3 and 4 but has retained exons 6 and 7. One caveat with this scenario is that this transcript also does not express exon 2. There are differences between the expression of these two exons between the two tissue types whereby the tumor has a mean relative intensity of 3.5 and the CELLs have a mean intensity of 2.9 indicating that this is the transcript expressed by the CELLs or there is another variant of this transcript not yet reported.

Figure 5b shows an example of intron retention in the UBXD5 gene. The tumor appears to be expressing sequences from the intron between exons 9 and 10. RefSeq annotates 3 representative transcripts, while H-InvDB annotates 15 different transcripts but none of these include retention of this intron. This provides further evidence that there are more transcripts occurring that are yet to be described. In support of this, a map of the expressed sequenced tag sites that map to this region show that a number of ESTs have been placed in the exact position of the retained intron shown in the inset of figure 5b.

Clearly, the data obtained from exon array analysis is very complex and requires in depth analysis of putative AS events. The availability of this data set which serves as a normal control in the analysis of colon diseases, will potentially help improve our understanding of the complex phenomenon of AS.

## Discussion

One of the challenges in whole transcript profiling has been to use the appropriate cell type in comparisons of tumor to normal CELLs. In this study, we present a dataset that we feel can serve as a normal control in the analysis of abnormal colon epithelium at the level of gene expression, and as a reference for AS profiles using the Affymetrix Exon1.0 ST arrays. The recent availability of arrays that allow the detection of AS events on a genome-wide scale, suggest that detection of gene expression at the transcript level alone may completely overlook gene expression changes at the level of splicing that are germane to the disease state.

The advantage of this particular data set stems from the fact that the profiles are derived exclusively from normal epithelial cells of the colon with no stromal contaminants. In colorectal cancer a gradual progression and accumulation of genetic alterations from the "normal"/non-neoplastic state to malignancy has been described. To date, it has not been possible to progressively propagate normal enterocytes in cell culture, making gene expression studies difficult [8]. Caco-2 cells have been used as normal controls for many studies, based on their "normal" appearance. The similarity to normal epithelial cells stems from phenotypic similarities to *in situ *enterocytes based on the acquisition of structural and functional polarity [9,10] but it has since been shown that expression characteristics similar to colon cancer have been observed in these cells [9]. Mucosa from fresh tissue specimens has also been used as a substitute for normal colonic epithelial cells [11-17], although microscopic examination of these samples clearly demonstrates that they represent a heterogeneous collection of cells with varying proportions of lympocytes scattered from the proximal to distal end of the colon. Clearly, these cells will compromise the analysis because of the contribution of the stromal components. In the latter, there is a preponderance of fibroblasts, the cells involved in the host's "desmoplastic" response to the invading tumor.

The approach described here has been previously proven to enrich for viable, unfixed colonic enterocytes and profiled them using the Affymetrix GeneChip EXON ST 1.0 array. We used the publicly available colon paired tumor/normal set to demonstrate the utility of this data. Analysis at the transcript level allowed a confirmation of genes that were differentially expressed between the tumor and normal tissues. This approach to the analysis of expression data may be an important application for this data set, as it verifies the gene expression changes in tissues while omitting those transcript alterations that are attributable to tissue heterogeneity. Of the 77 genes that showed expression alterations between the tumors compared to normal tissues and confirmed in the enterocytes, the most significant network identified was Cancer, Cell Growth and Proliferation and Cellular Movement. The majority of genes show interaction with TNF or TGFβ1 or both. Interestingly, both have been reported to be involved in the epithelial-mesenchymal transition (EMT) which characterizes the progression of many carcinomas, including colon cancer. Bates and Mercurio [18] demonstrated that TGFβ1-induced EMT is accelerated dramatically by the presence of activated macrophages, identified TNF as the critical factor produced by macrophages that accelerates the EMT in a model of colon cancer.

The EXON 1.0 ST array does not detect actual transcripts but the expression levels of individual exons, which are then reconstructed into transcripts virtually. The analysis does not provide information about which exons are co-expressed and in which orientation, but instead assembles genes based on the association of exons with that particular locus (Affymetrix Technical Note: Identifying and Validating Alternative Splicing Events: http://www.affymetrix.com/support/technical/technotes/id_altsplicingevents_technote.pdf). The common assumption is that expression levels derived from these whole-transcript arrays are more accurate predictors of expression. This is due to the fact that 3' arrays will typically miscall gene expression alterations in those genes that have alternative 3' splicing.

It is perhaps important to appreciate the advantages of using the Affymetrix GeneChip Exon ST 1.0 arrays and their ability to define AS. AS introduces several levels of complexity into the analysis of gene expression alterations, however, several key features have been identified. AS in a given gene is not an all-or-nothing event, and is usually a subtle shift in the actual levels of individual exon expression. Furthermore, error is introduced with variation in amplification efficiency across individual transcripts. Bemmo et al [19] suggest that the high GC content of 5' end, and reduced priming efficiency in the 3' end due to an artifact of random priming results in different signal intensities at the ends of genes and so increases the number of false positives. This can be further compounded by tissue heterogeneity and the fact that splicing patterns can be inherited [20]. Hybridization efficiency is also affected by the presence of SNP(s) within the sequence of the probeset [21]. AS has also been found to differ as a function of ethnicity [22]. One commonly used approach to the analysis of Exon array data is to use the Splicing Index, where the probeset intensity is divided by the entire transcript intensity (Affymetrix Technical Note: Identifying and Validating Alternative SplicingEvents: http://www.affymetrix.com/support/technical/technotes/id_altsplicingevents_technote.pdf). This approach suffers from some methodological and statistical drawbacks resulting in high false positive rates (see [19] for discussion). We used a very strict tactic for the detection of AS, to decrease the false positive rate but this approach may also eliminate possible AS events. The use of ANOVA, where the interaction between the sample type and probeset is used to indicate differential expression of probesets within the framework of whole transcripts, allows the removal of variables and permits a calculation of significance of AS in the absence of large fold changes at the transcript level. One caveat with this approach is that it assumes an unvarying response of each probeset within a transcript and this assumption is not met for probesets that are saturated or that hybridize weakly [19]. Using our stringent criteria for analysis we obtained a list of 497 presumed AS genes, although, upon visual examination of these genes, very few (~5%) actually exhibited convincing alternative splicing in genes with variants recorded in RefSeq, highlighting the need for careful examination of the data produced by such analyses. Most of the AS changes occurred in genes not reported to be alternatively spliced in RefSeq, and often not reported in more highly curated databases such as H-InvDB. Due to the large amount of EST and genomic information, a vast amount of information can be obtained from *in-silico *prediction of transcript isoforms but many of the available EST libraries are cloned from tumor samples and as such the databases tend to lack information on normal splice forms [23].

## Conclusions

Use of the Affymetrix GeneChip Exon ST 1.0 and Gene ST 1.0 arrays will provide an equitable and empirical platform to access information on transcript variants. This data set is a valuable asset for studies of colon disease. We have demonstrated that, using normalization across array and removal of variation attributed to technical processing, the data can be compared to tumors and normal tissue processed at a different time and place assuring that the data is transportable. The raw data is available at GEO.

## Methods

### Sample Processing

Specimen procurement was approved through the Health Institutional Review Board at the State University of New York at Buffalo. Specimens were obtained after surgical removal, and cells obtained from non-diagnostic, excess areas of tissue. Tissue was not collected specifically for the described research. The samples were de-identified and the researchers had no contact with the human subjects.

Briefly, the procurement protocol involved receipt of the extirpated specimen in the operating suite, rapid transport to the pathology department, opening and gross inspection of the specimen and removal of debris with normal saline washes warmed to body temperature, followed by exfoliation of cells with the edge of a glass slide [24]. The exfoliated cells were then placed into a microcentrifuge tube containing PBS with 10 mM dithiothreitol (DTT), a mucolytic agent also warmed to body temperature. During the development of this procurement protocol, elimination of mucus was found to be necessary to prevent contamination by symbiotic bacteria present in the human intestinal tract and other cells that may have become trapped in the mucus during the exfoliation procedure. The groups of cells that were exfoliated were further dispersed into single and small groups of cells using a chelating agent (Cellstripper™, Mediatech, Herndon, VA). These washes were then followed by further enrichment with a red blood cell lysis agent (RBC Lysis Buffer, eBioscience). Final enrichment was achieved using magnetic beads coated with the ber-Ep4 antibody, which recognizes an epitope previously documented to be expressed in colonic epithelial cells, which is considered to be specific for this cell type (Gaffey et al, 1992). The enriched cells were snap frozen in liquid nitrogen and stored at -80°C. Total RNA from each sample was extracted within one month of procurement

**Figure 6:**
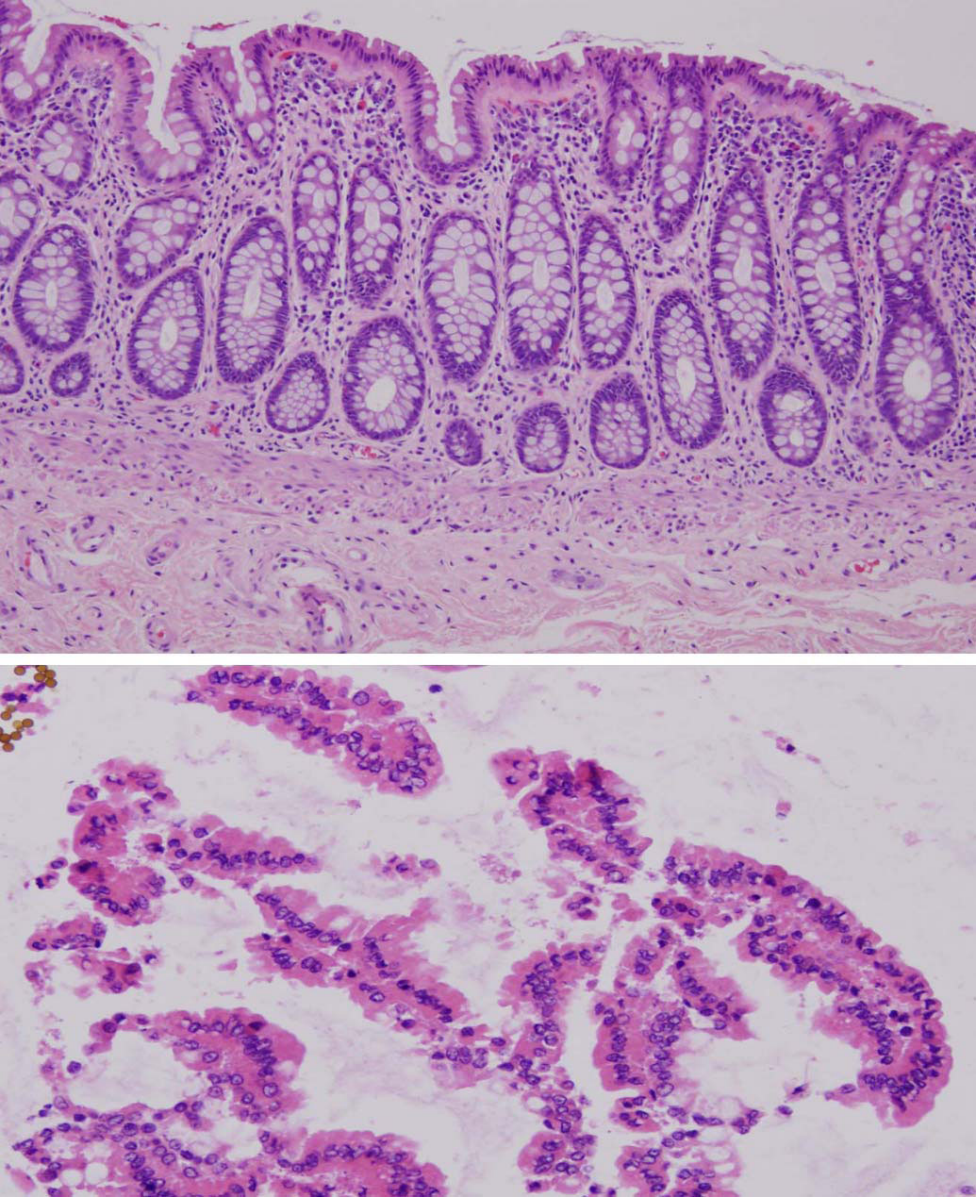
**Histology of colonic mucosa**. (1a) Section of mucosa was stripped off the colectomy specimen and processed by formalin fixation and paraffin embedding. The mucosa is composed of the lamina propria and epithelium. As is evident, it is not a homogeneous collection of cells, but rather a composite of the cells that are present in the lamina propria (chronic inflammatory cells, smooth muscle cells and vessels) and epithelium (hematoxylin and eosin 10×). (1b) Histologic evidence of enrichment for colonic epithelial cells from the procurement approach described in the text (hematoxylin and eosin 20×).

To control for variables associated with specimen integrity and sample processing, the time intervals between receipt of the sample in the operating suite and extirpation of each specimen was recorded. Additionally, the time intervals for delivery to the laboratory, placement of exfoliated cells in PBS with DTT, and the endpoint wherein enriched cells were stabilized by snap freezing were also documented and presented in table 3. A portion of the exfoliated cells from one case were processed in parallel to those procured for microarray studies, but retained and fixed in 1% glutaraldehyde. These cells were then concentrated and the pellet used to generate a cell block, which was processed, sectioned and then stained for microscopic examination. From this same colectomy specimen, a small strip of colonic mucosa was also procured, fixed, processed and stained for comparative purposes to document cellular heterogeneity (figure 6).

**Table 3:** Sample Processing Variables

Case #	TI #1 (min)	TI #2 (min)	TI#3 (min)	TI#4 (min)	TI#5 (min)
1	5	3	2	41	51

2	5	3	2	41	51

3	3	2	2	42	49

4	1	2	7	44	54

5	1	1	3	41	46

6	1	3	3	41	48

7	2	2	2	40	46

8	2	2	3	39	46

9	2	3	3	52	60

10	1	2	2	44	49

The procurement protocol sought to enrich the targeted cell population in as short and consistent time frame to reduce the possibility of introducing external bias. Time intervals (TI) for the various processing methods of the collected samples. TI#1: Time interval between extirpation and receipt of specimen. Differences in times were due to different surgeons. TI#2 Time interval between receipt of specimen and transit to Department of Pathology. TI#3 Time interval between opening of the specimen, exfoliation of the cells and placement into PBS; TI#4 Time interval between placement of cells in PBS and freezing of cells in liquid nitrogen. TI#5: Time interval summary for the entire procurement process.

### Exon Array

The GeneChip^® ^Human Exon 1.0 ST (sense target) array is a whole-genome array, containing over 1.4 million probesets of up to four perfect match (PM) probes each, spread across exons from all known genes, plus a number of additional regions based on other annotation sources, including GENSCAN predictions and ESTs from dbEST. In the design phase, sequences from all the annotation sources were mapped to the July 2003 version of the human genome (UCSC hg16, NCBI 34).

The array contains only PM probes, designed from NCBI Build 35, with a small number of generic mismatch probes for background correction. No probes span exon-exon junctions (Affymetrix Exon Probeset Annotations White Paper http://www.affymetrix.com/support/technical/whitepapers/exon_probeset_trans_clust_whitepaper.pdf). Each probeset is assigned to a 'transcript cluster', and also has an annotation quality indicator associated with it.

Using a random hexamer incorporating a T7 promoter, double-stranded cDNA was synthesized from 500 ng total RNA from which the majority of the ribosomal RNA had been removed using a RiboMinus Human/Mouse Transcriptome Isolation Kit (Invitrogen, Carlsbad, CA). cRNA was generated from the double-stranded cDNA template though an in-vitro transcription reaction and purified using the Affymetrix sample cleanup module. cDNA was regenerated through a random-primed reverse transcription using a dNTP mix containing dUTP. The RNA was hydrolyzed with RNase H and the cDNA purified. The cDNA was then fragmented by incubation with a mixture of UDG and APE 1 restriction endonucleases; and end-labeled via a terminal transferase reaction incorporating a biotinylated dideoxynucleotide. *5.5 *ug of the fragmented, biotinylated cDNA was added to a hybridization cocktail, loaded on a Human Exon 1.0 ST GeneChip and hybridized for 16 hours at 45°C and 60 rpm. Following hybridization, the array was washed and stained according to the Affymetrix protocol. The stained array was scanned at 532 nm using an Affymetrix GeneChip Scanner 3000, generating CEL files for each array.

### Gene Expression Analysis

Several different comparisons were performed for gene expression alterations; i) tumor compared to normal tissues from the downloaded data set (TUMOR VS NORMAL), ii) tumor compared to the isolated colon epithelial cells (TUMOR VS CELLS) and iii) normal tissue compared to isolated epithelial cells (NORMAL VS CELLS). Gene expression alterations were determined using PARTEK Genomics Suite Software. The .CEL files were imported from the Affymetrix Expression Console and background correction, normalization and probe summarization was performed using RMA. Principle component (PCA) analysis was performed on both sets of data to examine whether clusters could be explained by the first few principle components. PCA is used for dimensionality reduction by retaining the characteristics of the data that contribute most to its variance.

A gene summarization is then performed on the data which estimates the intensity of individual genes by averaging the intensities of all the probsets comprising the gene. The summarization is followed by a 5-way analysis of variance (ANOVA) using a mixed model and methods of moment to equate ANOVA mean sum of squares to their expected values. Due to the fact that each transcript on the EXON 1.0 ST array has multiple measurements, the ANOVA model incorporates a variance measurement of exon-to-exon differences. Other variables included in this analysis were; scan date, age, sex and tissue type. Undesired batch effects due to processing from different laboratories were removed and the data reanalyzed using a two sample t-test for significance at p = 0.05 and a fold change cutoff of 2.

To assess the possible functional connections between the differentially expressed genes (DEGs), a pathways analysis, which assesses statistically overrepresented functional terms within a list, was conducted using Ingenuity Pathways Analysis (IPA) for all comparisons. The probability that a specific set of genes has a significant number of members in a canonical pathway is assigned a p-value which is calculated by Fisher's Exact Test (right tailed). This p-value is based upon the number of genes in the pathway, the number of focus genes in each comparison list that belong to the pathway and the number of corresponding number of genes in each comparison that represent inputs. The p-value indicates the probability of observing the fraction of the focus genes in the canonical pathway compared to the fraction expected by chance in the reference set, with the assumption that each gene is equally likely to be picked by chance.

IPA generates networks based on the input eligible molecules in the DEG list. The score is a numerical value used to rank the generated networks according to their degree of relevance to the molecules represented by the DEG list. The score is generated using a right-tailed Fischer's Exact test and the score is the negative log of this p value. Functional analysis of the three DEG lists was also performed using IPA. This enables an association of biological functions with the DEG list using the Ingenuity knowledge database.

### Splice Variant Analysis

Exon-level expression values were derived from the CEL file probe-level hybridization intensities using the model-based RMA algorithm as implemented in the Partek Genomics Suite. For this analysis we used the Core meta-probeset which are the most conservative set of genes predicted to have the highest confidence score for transcript clusters. The Core set contains 230,000 exon probesets that have been mapped to 17,800 empirically supported core transcripts.

A two-way ANOVA was performed to compare tumors to isolated epithelial cells, with "scan date" as an ANOVA factor and "tissue type" as the alternative splice factor. A lack of expression can be mistaken for alternative splicing because low signals mean that the array measures experimental noise. For this reason, exons that were not present in at least one group were removed from the analysis by restricting the inclusion of probesets to those that have a higher relative signal intensity of 3. In order to differentiate the exons showing significant differences that were alternative splice events from those that were whole transcripts with significant expression differences, we assigned an empirically determined filter such that the exon-specific probesets required a p value for alternative splicing of <0.0001, the p value for whole transcript expression was >0.9 and the fold change cut off for whole transcript expression was <2 and >-2.. In this way we have eliminated those exons whose expression changes between the tumor and normal cells is due to the whole transcript differential expression. The individual exon expression values were examined visually in the context of the whole transcript using Partek Genomics Suite; Gene View. In each of the reported cases, the exon sequence was "blasted" to determine if the changes seen could be attributed to cross hybridization. We also limited our reporting of AS events to alternatively spliced genes that were recorded in RefSeq because we had no way to validate the data and the complexity of this type of data makes it highly susceptible to false discoveries (see Discussion).

## Authors' contributions

WM procured the samples under IRB approved protocols. He collected samples from the operating suite and performed subsequent epithelial cell enrichment. He assessed the purity of the samples and aided in study design and manuscript preparation. LH designed the study, performed the analysis and drafted the manuscript. All authors read and approved the final manuscript.

## Supplementary Material

Additional file 1 **Wnt-Signaling Pathway Identified by Comparison of Tumor v Cells**. Ingenuity Pathways Analysis of Tumor v Cells differentially expressed genes (DEG List). IPA identified this canonical pathway as having a significant number of genes associated with it from the DEG list. The genes shown in red are up-regulated in the DEG list and those in green are down-regulated in the DEG list. The more intense colors represent larger fold change differences. The solid lines between the molecules indicate the source molecule up-regulates the target molecule and the dashed lines indicate down-regulation. Click here for file

Additional file 2 **Network Generated by DEG list of Tumor vs Cells**. This network, identified by the tumor vs cell comparison involves Cancer Cell Cycle, Cellular Growth and Proliferation functions and has a score of 20 (p = 10E-20) and has 35 molecules fitting into the network. It is apparent from the network ideogram that all molecules interact either directly or indirectly through the CDKN1A gene. Red indicates increased expression and green indicates decreased expression. The solid lines between the molecules indicate the source molecule up-regulates the target molecule and the dashed lines indicate down-regulation. Click here for file

Additional file 3 **Network Generated by DEG list of Tumor vs Normal Tissues**. This network, identified by the tumor vs normal tissue comparison involves Cancer, Cell Movement, Hematological System Development and function. The score for this network was has a score of 16 (p = 10E-16). It is apparent from the network ideogram that all molecules interact either directly or indirectly through the TNF or IL1B genes, although these genes themselves do not show altered expression in the tumors compared to normal tissues Red indicates increased expression and green indicates decreased expression. The solid lines between the molecules indicate the source molecule up-regulates the target molecule and the dashed lines indicate down-regulation. Click here for file

Additional file 4 **Network Generated by the DEG list of Tumor vs Cells/Normal Overlap**. This network, was identified by the tumor vs cells and normal tissue comparison (list of 77 genes). The network identified involves Cancer, Cell Growth and Proliferation, Cellular Movement and has an associated score of 30 (p = 10E-30). The majority of the genes showing down-regulation are interacting with IL8, TNF and TGFB1, while genes showing increased expression show direct interactions with HNF1A. Red indicates increased expression and green indicates decreased expression. The solid lines between the molecules indicate the source molecule up-regulates the target molecule and the dashed lines indicate down-regulation. Click here for file

Additional file 5 **List of Alternative Splice Events**. A strict method of detecting AS was developed, which was designed to decrease the false positive rate, although it may also have eliminated some possible AS events. We also limited our analysis to a comparison between the tumors and isolated epithelial cells (CELLs). The exon-specific probesets required p values for alternative splicing of <0.0001, the p value for whole transcript expression was >0.9 and the fold change cut off for whole transcript expression had to be <2.5 and >-2.5. In this way we eliminated whole transcript expression while detecting exon-specific changes and defined 497 genes showing alternative splicing. Click here for file
