# Supplementary figures and images for: Normal colon epithelium: a dataset for the analysis of gene expression and alternative splicing events in colon disease
Source: BMC Genomics. 2010 Jan 4;11:5. doi: 10.1186/1471-2164-11-5 (PMC2823691; doi:10.1186/1471-2164-11-5)

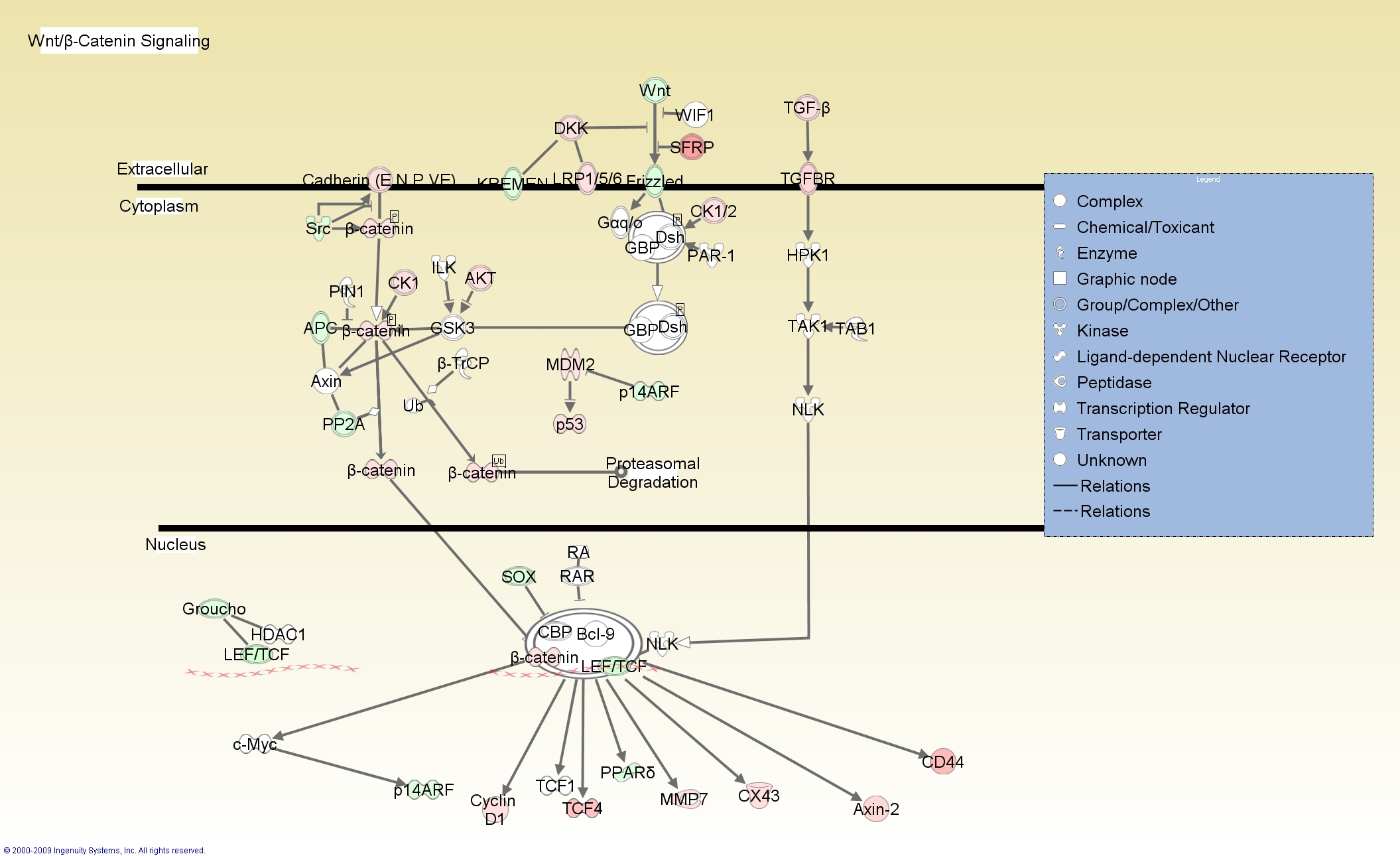

Supplement: Additional file 1 — Wnt-Signaling Pathway Identified by Comparison of Tumor v Cells. Ingenuity Pathways Analysis of Tumor v Cells differentially expressed genes (DEG List). IPA identified this canonical pathway as having a significant number of genes associated with it from the DEG list. The genes shown in red are up-regulated in the DEG list and those in green are down-regulated in the DEG list. The more intense colors represent larger fold change differences. The solid lines between the molecules indicate the source molecule up-regulates the target molecule and the dashed lines indicate down-regulation. [file 1471-2164-11-5-S1.JPEG]
